# Reconstruction of the kinetochore: a prelude to meiosis

**DOI:** 10.1186/1747-1028-2-17

**Published:** 2007-06-06

**Authors:** Haruhiko Asakawa, Tokuko Haraguchi, Yasushi Hiraoka

**Affiliations:** 1 Kansai Advanced Research Center, National Institute of Information and Communications Technology, 588-2 Iwaoka, Iwaoka-cho, Nishi-ku, Kobe 651-2492, Japan; 2 Department of Biology, Graduate School of Science, Osaka University, 1-1 Machikaneyama, Toyonaka 560-0043, Japan; 3 Graduate School of Frontier Bioscience, Osaka University, 1-3 Yamadaoka, Suita 565-0871, Japan

## Abstract

In eukaryotic organisms, chromosomes are spatially organized within the nucleus. Such nuclear architecture provides a physical framework for the genetic activities of chromosomes, and changes its functional organization as the cell moves through the phases of the cell cycle. The fission yeast *Schizosaccharomyces pombe *provides a striking example of nuclear reorganization during the transition from mitosis to meiosis. In this organism, centromeres remain clustered at the spindle-pole body (SPB; a centrosome-equivalent structure in fungi) during mitotic interphase. In contrast, during meiotic prophase, centromeres dissociate from the SPB and telomeres cluster to the SPB. Recent studies revealed that this repositioning of chromosomes is regulated by mating pheromone signaling. Some centromere proteins disappear from the centromere in response to mating pheromone, leading to dissociation of centromeres from the SPB. Interestingly, mating pheromone signaling is also required for monopolar orientation of the kinetochore which is crucial for proper segregation of sister chromatids during meiosis. When meiosis is induced in the absence of mating pheromone signaling, aberrant chromosome behaviors are observed: the centromere proteins remain at the centromere; the centromere remains associated with the SPB; and sister chromatids segregate precociously in the first meiotic division. These aberrant chromosome behaviors are all normalized by activating the mating pheromone signaling pathway. Thus, action of mating pheromone on the centromere is important for coherent behavior of chromosomes in meiosis. Here we discuss repositioning and reconstruction of the centromere during the transition from mitosis to meiosis, and highlight its significance for proper progression of meiosis.

## Background

Eukaryotic chromosomes are spatially organized within the nucleus. While such nuclear architecture provides a physical framework for the genetic activities of chromosomes, this framework however is dynamic, able to change its functional organization during the cell cycle or developmental stages. Local chromatin structures change as chromosomes undergo processes such as replication, transcription, recombination and repair. During chromosome segregation, a specialized structure called kinetochore is formed on the centromeric DNA. Global organization of chromosomes within the nucleus can also change in association with their activities. A prominent example of reconstruction of the nuclear and chromosomal frameworks is observed during the transition from mitosis to meiosis.

Meiosis is a process that produces haploid gametes from parental diploid germ cells in sexually reproducing organisms. In this process, a single round of chromosome replication is followed by two rounds of chromosome segregation. A characteristic feature of meiosis is the behavior of sister chromatids in the first division (meiosis I). In meiosis I, homologous chromosomes segregate while sister chromatids remain held together. Sister chromatids then segregate in the second division (meiosis II). Reductional segregation of homologous chromosomes in meiosis I requires a recombination-mediated physical link between a pair of homologous chromosomes. This regulated segregation of homologous chromosomes and sister chromatids is achieved by selective use of monopolar and bipolar structures of the kinetochore, which provide a structural basis for monopolar and bipolar attachment of the spindle, respectively. Monopolar attachment of the spindle to sister kinetochores leads to movement of sister chromatids to the same pole in meiosis I. Bipolar attachment of the spindle to sister kinetochores causes segregation to the opposite poles in meiosis II (as well as in mitosis).

Meiotic reorganization of the chromosome is associated with assembly of the meiotic chromatin proteins that replace the mitotic counterparts. Cohesin proteins are among such proteins, and affect structures and segregation patterns of chromosomes [1,2]. The position of centromeres in the nucleus also changes during the transition from mitosis to meiosis. In mitotic interphase, chromosomes are organized in a polarized orientation, with centromeres confined near the centrosome and telomeres positioned at the opposite side of the nucleus (often called the Rabl orientation). In contrast, in meiotic prophase, telomeres become clustered beneath the nuclear envelope near the centrosome; this arrangement of chromosomes bundled at the telomeres is often called the bouquet arrangement because of its shape (reviewed in [3]). The fission yeast *Schizosaccharomyces pombe *provides a striking example of the repositioning of centromeres and telomeres upon entering meiosis. Studies on regulatory mechanisms of this phenomenon have elucidated important aspects of centromere reconstruction that may influence the progression of meiosis.

## The fission yeast meiosis: an overview

**Figure 1:**
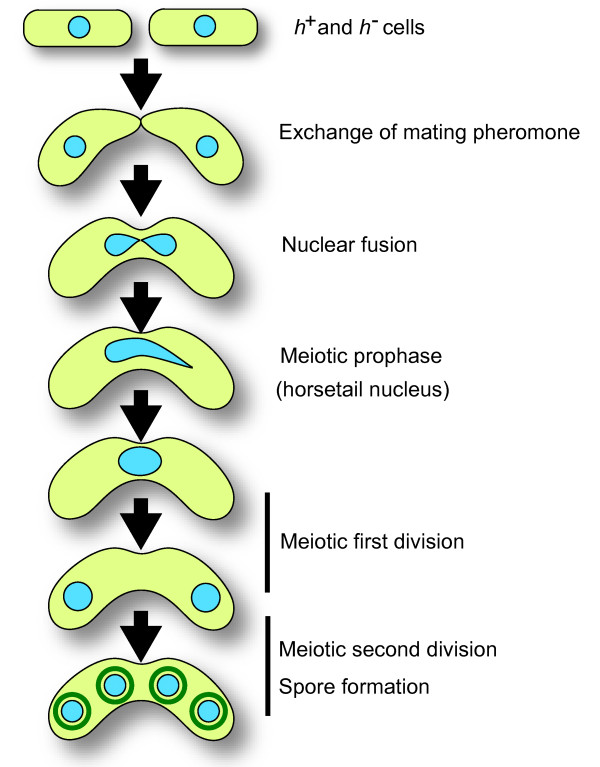
**The meiotic process in *S. pombe***. When starved of nitrogen sources, haploid cells of the *h*^+ ^and *h*^- ^mating type are arrested in G1, and secrete the mating pheromone to the opposite mating type cells. Upon sensing the pheromone, the cell elongates toward the opposite mating type cell and the cells fuse with each other (conjugation), followed by fusion of the haploid nuclei. The fused diploid nucleus then elongates (known as the horsetail nucleus), and moves back and forth between the cell ends (meiotic prophase). Meiotic DNA replication occurs at the beginning of this period, yielding a nucleus with 4C DNA contents. After movement ceases, the horsetail nucleus becomes rounded at the center of the cell, and proceeds with the first and second meiotic divisions to create four 1C nuclei.

The fission yeast *S. pombe *is a unicellular eukaryotic organism. This yeast usually grows as haploid cells with a genome of three chromosomes. Meiotic processes of *S. pombe *are summarized in Figure 1. When cells are starved for nitrogen sources, they arrest their cell cycle in G1 phase and secrete mating pheromone. Cells of the opposite mating type (*h*^+ ^and *h*^-^) exchange mating pheromones, and subsequently conjugate to form a diploid zygote. In the diploid zygote, *h*^+ ^and *h*^- ^haploid nuclei fuse to form a diploid nucleus. Immediately after nuclear fusion the nucleus elongates and moves back and forth between the cell poles. The elongated nucleus is often called the "horsetail" nucleus because of its shape. Meiotic DNA synthesis and recombination occur in this horsetail nucleus. After nuclear movement, the cell undergoes meiotic divisions, and the nucleus is divided into four haploid nuclei. Each nucleus is encapsulated within a spore after meiosis II.

**Figure 2:**
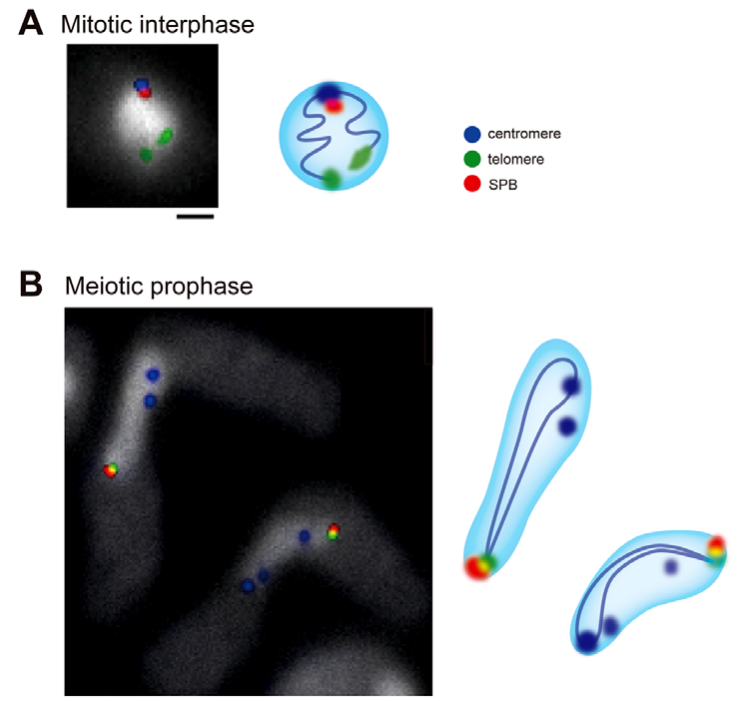
**Spatial organization of *S. pombe *chromosomes**. Location of centromeres and telomeres in mitotic interphase (A) and meiotic prophase (B) determined by FISH analysis using specific probes. The SPB was detected by indirect immunofluorescence. Scale bar, 1 μm. Right figures are schematic models of spatial organization of the nucleus. The blue line superimposed on the nucleus illustrates a chromosome.

Significantly, centromeres and telomeres switch their positions in the nucleus upon entering meiosis [4,5]. Before meiosis, centromeres are clustered at the spindle-pole body (SPB, an equivalent structure to the animal centrosome), and telomeres are attached to the nuclear membrane, showing a typical Rabl orientation. In contrast, upon entering meiosis, telomeres cluster to the SPB and centromeres become dissociated from the SPB, showing the bouquet configuration (Figure 2). In nuclear fusion, haploid nuclei approach each other, with the telomere-SPB cluster located at the leading edge of each nucleus. After nuclear fusion, telomeres remain clustered at the SPB located at the leading edge of the elongated nucleus moving between the poles. Repositioning of centromeres and telomeres is observed in meiotic cells in many eukaryotes from yeasts to plants and animals including humans [6-9].

## Telomeres are clustered in response to mating pheromone signaling

While telomere clustering in meiotic prophase has been observed in a wide variety of organisms, and its role in meiosis has been inferred (reviewed in [10]), analyses in *S. pombe *have demonstrated that telomere clustering and nuclear movement promote homologous chromosome pairing [11,12]. In *S. pombe*, telomere clustering is known to take place in response to mating pheromone [13], and recently the underlying molecular mechanism has been revealed[14]. *S. pombe *Taz1 (an ortholog of the human TRF1 and TRF2) binds directly to telomeric DNA, and Rap1 binds to the telomere through interaction with Taz1 [15-17]. During the mating pheromone response, meiosis-specific proteins Bqt1 and Bqt2 are expressed and the Bqt1/Bqt2 complex recruits Sad1 (an SPB component) to Rap1 at the telomere [14]. In the presence of Bqt1 and Bqt2, Sad1, which is usually localized at the SPB, becomes accumulated at the scattered telomeres to form multiple foci on the nuclear envelope, and subsequently the Sad1 foci converge to the SPB together with telomeres. In this process, Sad1 binds to another SPB component Kms1, which interacts with the dynein motor protein [18,19], and Sad1-bound telomeres are then clustered to the SPB through interaction with the dynein motor complex along microtubules. Horsetail nuclear movement is also mediated by cytoplasmic microtubules and the dynein motor complex [19-21]. Thus, telomeres are clustered to the SPB via the meiosis-specific connector proteins Bqt1 and Bqt2 in response to mating pheromone signaling, and remain connected to the SPB throughout the horsetail nuclear movement.

## Centromeres are disassembled in response to mating pheromone signaling

**Figure 3:**
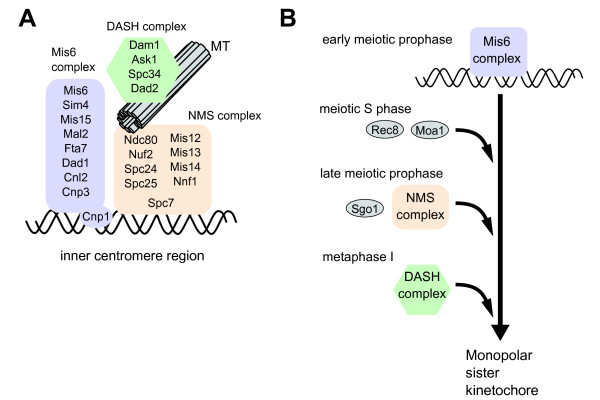
**Dynamics of centromere proteins during meiosis**. (A) *S.pombe *centromere protein complexes. Three major complexes are located at the inner centromere region during mitosis. The NMS complex consists of the Ndc80 complex (Ndc80, Nuf2, Spc24, and Spc25), the Mis12 complex (Mis12, Mis13, Mis14, and Nnf1), and Spc7. The Ndc80 complex and the DASH complex are required for kinetochore-microtubule attachment in mitosis. The DASH complex, forming a ring structure, binds to and slides along the spindle microtubules. In mitotic interphase, the DASH complex does not localize to the centromere region. (B) Reassembly of centromere proteins during meiotic prophase. In early meiotic prophase, the NMS and DASH complex proteins are dissociated from the centromere while the Mis6 complex remains. Meiosis-specific cohesin Rec8 and the Rec8-associated protein Moa1 are recruited during meiotic DNA replication. In late meiotic prophase, Sgo1 and NMS complex proteins are assembled onto the centromere. Finally, DASH complex associates with the centromere upon metaphase I.

Centromeres are separated from the SPB during meiotic prophase in *S. pombe*. Recent studies have revealed that a group of proteins disappear from the centromere during meiotic prophase, regulated by mating pheromone signaling [22,23]. In mitotic interphase, a number of proteins assemble at the centromere and connect the centromere to the SPB; when the cell enters mitosis, they form the kinetochore as an interface between the centromere and spindle microtubules. As in other eukaryotic organisms, the *S. pombe *kinetochore is composed of several subcomplexes of centromere proteins: three centromere subcomplexes named Mis6, NMS (Ndc80-Mis12-Spc7), and DASH complexes [24,25]. The Mis6 complex and NMS complex remain at the centromere throughout the mitotic cell cycle, while the DASH complex locates to the centromere shortly before metaphase [23,25] (Figure 3A). These complexes have distinct localization patterns during meiosis. Upon entering meiosis, the NMS complex disappears from the centromere, whilst the Mis6 complex keeps its location at the centromere [22,23] (Figure 3B). Removal of the NMS complex induces dissociation of centromeres from the SPB [22]. In late prophase, the NMS complex reappears at the centromere, and the DASH complex appears shortly before metaphase of meiosis I. These observations suggest that the Mis6 complex constitutes the structural basis of the centromere and that the NMS and DASH complexes reassemble to establish the functional metaphase kinetochore, together with meiosis-specific factors [22,23] (Figure 3B, see below). Meiosis-specific removal and recovery of the NMS complex implies a role associated with reconstruction of the kinetochore. Disassembly and reassembly of the centromere proteins might be prerequisite events for construction of the meiotic monopolar kinetochore, although this remains to be proven.

## Monopolar sister kinetochore is formed in response to mating pheromone signaling

Studies of meiosis in *S. pombe *have revealed that centromere dissociation from the SPB and formation of the monopolar sister kinetochore are both regulated by mating pheromone signaling. This correlation was first suggested by analyses using the *pat1 *mutant [26,27]. Pat1 kinase is a key negative regulator of meiosis. In wild-type cells, mating pheromone signaling is required to inactivate Pat1 upon meiosis initiation. This requirement for mating pheromone signaling can be bypassed by inactivation of Pat1 by temperature-sensitive mutation. Mutational inactivation of Pat1 induces cells to enter meiosis even under conditions that the cell normally never enters meiosis, e.g., in the haploid state [28,29]. In these experiments, cells are arrested in G1 phase by nitrogen starvation prior to induction of meiosis. Interestingly, in cells induced into meiosis by mutational inactivation of Pat1, in the absence of meting pheromone signaling, telomeres are clustered to the SPB, but centromeres often remain at the SPB in meiotic prophase [27]. In those cells, the NMS complex localizes to centromeres attached to the SPB [22,23]. Furthermore, sister chromatids are precociously segregated to opposite poles in meiosis I, indicating a deficiency in monopolar attachment of the sister kinetochores [26].

Meiotic defects caused by mutational inactivation of Pat1 are due to the lack of mating pheromone signaling. When the *pat1 *mutant cells are induced to meiosis in the presence of mating pheromone signaling, NMS complex proteins disappear from centromeres and centromeres are fully dissociated from the SPB [22,23]. Moreover, sister chromatids correctly moves to the same spindle pole, indicating the monopolar sister kinetochore has been established [26]. These findings demonstrate that the monopolar sister kinetochore is formed in response to the mating pheromone, causing reorganization of centromere proteins in meiotic prophase.

As the sister kinetochore is constructed during DNA replication, it is possible that the timing of DNA replication is affected by the presence or absence of mating pheromone. The timing of meiotic DNA synthesis can be determined in meiosis synchronously induced by temperature shifting G1 cells of the *pat1 *mutant. When meiosis is induced by *pat1 *mutation without mating pheromone, DNA synthesis occurs after 4 hours; on the other hand, when meiosis is induced in the presence of mating pheromone signaling, DNA synthesis occurs after 2 hours [26]. Thus the mating pheromone signaling may also induce meiotic S-phase earlier. Importantly, in the *pat1 *mutation-induced meiosis without mating pheromone, the NMS complex remains at the centromere, and centromeres often remain associated with the SPB at the time of meiotic S-phase [22,23,27]. In contrast, in meiosis induced by the mutational inactivation of Pat1, in the presence of the mating pheromone signaling, the NMS complex disappears from the centromere, and centromeres are dissociated from the SPB before meiotic S-phase like in the normal process of meiosis [22,23]. These results suggest that formation of the monopolar kinetochore correlates with removal of the NMS protein complex and consequent separation of centromeres from the SPB at the time of DNA replication, although no evidence has been found to support direct causality.

## Centromere loading of Sgo1, but not of Rec8 or Moa1, depends on mating pheromone signaling

Some meiosis-specific proteins are known to play a role in the monopolar attachment of sister kinetochores in *S. pombe*. Meiotic cohesin Rec8 holds sister kinetochores together until anaphase II [30]. Along with Rec8, Moa1 is essential in establishing the monopolar kinetochore [31]. During meiotic S-phase, Rec8 is loaded onto the sister chromatids [32]. Moa1 is loaded to the centromere in an early stage of horsetail nuclear movement [23], probably corresponding to S-phase similarly to Rec8 (Figure 3B). The loading of Rec8 and Moa1 does not require mating pheromone signaling [23,26]. In addition, Rec8 and Moa1 can locate to the centromere when ectopically expressed in mitotic interphase cells [31,33], while the NMS complex remains bound to the centromere. Thus, the removal of the NMS complex may be unnecessary for loading of Rec8 and Moa1 to the centromere. Monopolar sister kinetochore formation further requires mating pheromone signaling, suggesting that centromere loading of Rec8 and Moa1 is necessary, but not sufficient for formation of the monopolar kinetochore. Additional alteration of the kinetochore may occur in response to the mating pheromone signaling.

Localization of another meiosis-specific protein, Sgo1, to the centromere is regulated by mating pheromone signaling. Sgo1 protects Rec8 at the centromere to maintain cohesion between sister kinetochores until meiosis II [34]. Sgo1 appears at the centromere during late-stage horsetail nuclear movement, as does the NMS complex [23] (Figure 3B). Importantly, when meiosis is induced by *pat1 *mutation in the absence of mating pheromone signaling, loading of Sgo1 to the centromere is severely diminished [23]. This suggests that loading of Sgo1 is regulated downstream of mating pheromone signaling. Rec8 is expressed in *pat1 *mutation-induced meiosis in the absence of mating pheromone signaling as well as normal meiosis in wild type cells. Because Sgo1 is capable of localizing to centromeres in proliferating cells when ectopically coexpressed with Rec8 [34], diminished localization of Sgo1 in the *pat1 *mutant might be due to insufficient expression. Details of the mechanism of Sgo1 regulation by mating pheromone signaling are yet to be elucidated.

## Regulatory pathways downstream of the mating pheromone

**Figure 4:**
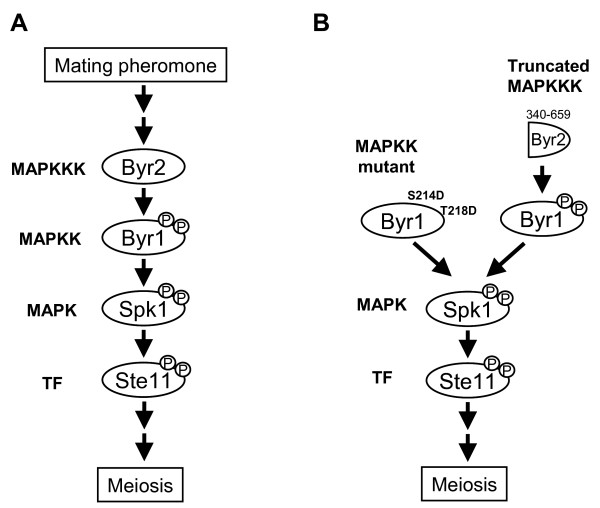
**Mating pheromone signaling pathway in *S. pombe***. (A) MAP kinase cascade of the S. pombe mating pheromone signaling pathway consists of Byr2 (MAPKKK), Byr1 (MAPKK), and Spk1 (MAPK). Spk1 phosphorylates and activates downstream transcription factor, Ste11. (B) Induction of meiosis by activation of the pheromone-responsive MAPK cascade. Expression of a mutant version of Byr1 (an analog form of the activated Byr1, in which serine at residue 214 and threonine at residue 218 are substituted to aspartic acids) can induce meiosis ectopically via activation of Spk1. Expression of a truncated version of Byr2 (residue 340–659) lacking the N-terminal regulatory domain can also activate downstream Byr1 and induce meiosis.

The previous sections describe the role of mating pheromone signaling in regulation of centromeres during meiosis. Moreover, ectopic activation of mating pheromone signaling can induce the normal process of meiosis. The mating pheromone signal is transmitted through a MAPK cascade, which consists of Byr2 (MAPKKK), Byr1 (MAPKK), and Spk1 (MAPK) [35-40]. This pheromone-responsive MAPK signaling cascade can be activated by expressing a mutant Byr1 protein that mimics a phosphorylated form of the molecule [41], or by expressing a truncated Byr2 lacking the N-terminal regulatory domain [42] (Figure 4). When the MAPK cascade is ectopically activated in cells, the chromosomes behave as in the normal process of meiosis: telomeres cluster to the SPB and centromeres dissociate from the SPB, one of the NMS complex proteins, Nuf2, is removed from the centromere in those cells, suggesting the entire NMS complex is probably removed from centromeres [41] (HA and YH, unpublished result), and while the sister chromatids show monopolar attachment to the spindle in meiosis I [41]. These facts indicate that chromosomal events proceed normally in meiosis induced by activated mating pheromone signaling [41]. These events require functional MAPK Spk1, because they do not occur in Spk1 mutant cells [41,42] (HA and YH, unpublished result). Thus mating pheromone signaling can induce meiosis with normal regulated behaviors of chromosomes.

Recently, a transcription factor, Ste11, was reported to function downstream of Spk1 [42]. Ste11, originally identified as an HMG family transcription factor, can bind to a specific sequence upstream of target genes [43]. Ste11 is primarily expressed upon nitrogen starvation, and expression is further increased in response to mating pheromone [42]. Spk1 phosphorylates two threonine residues of Ste11 (residues 305 and 317) *in vitro*, and phosphorylation of these residues is required for haploid meiosis induced by ectopic activation of the MAPK cascade [42]. Thus, factors regulated by Ste11 may be involved in meiotic centromere reconstruction. Alternatively, unknown factors that are directly phosphorylated by Spk1 may be involved. Recent DNA microarray analyses have identified more genes whose expression is induced by mating pheromone signaling [14,44,45]. A more comprehensive search of factors downstream of mating pheromone signaling will lead to further understanding of meiotic regulation of the centromere.

## Regulation of centromeres in other organisms

Several pieces of evidence for centromere-telomere repositioning and regulation of centromere proteins in the transition from mitosis to meiosis have been reported in other eukaryotes. In the budding yeast *Saccharomyces cerevisiae*, repositioning of chromosomes takes place during meiosis, although the telomere clustering is less prominent and shorter in time than that in *S. pombe *[8,46]. At the time of repositioning, one of the Ndc80 complex proteins, Nuf2, disappears from the centromere, suggesting that the repositioning probably involves reorganization of centromere proteins as in *S. pombe *[22]. In *S. cerevisiae*, proteins crucial for monopolar attachment of sister kinetochores to the spindle have been identified [47-53], but the correlation with spatial regulation of centromeres has not been examined. It is unlikely that the mating pheromone response regulates monopolar sister kinetochore formation because although the mating pheromone induces cell fusion, it does not induce meiosis directly in this organism.

In the nematode *Caenorhabditis elegans*, spatial reorganization of the nucleus occurs at the onset of meiotic prophase. Chromosomes asymmetrically cluster to one side of the nucleus, which in turn becomes crescent-shaped [54], although it is unclear whether reorganization of chromosomes involves repositioning of centromeres and telomeres. In *C. elegans*, several centromere proteins such as Cenp-C, Him-10 and KNL-1 are localized only during chromosome segregation both in mitosis and meiosis [55-59]. Remarkably, histone H3 variant, Cenp-A, loses the localization selectively during meiotic prophase, while it remains at the centromere throughout the mitotic cell cycle [55,60]. It is not known, however, whether the disappearance of these proteins is related to meiotic reorganization of the nucleus and monopolar kinetochore formation in *C. elegans*.

In higher eukaryotes, reorganization of the nucleus occurs during meiosis [6]. A large number of kinetochore proteins, including homologues of *S. pombe *proteins, has been reported. However, it is largely unknown how the kinetochore proteins behave during meiosis in higher eukaryotes.

## Conclusion

**Figure 5:**
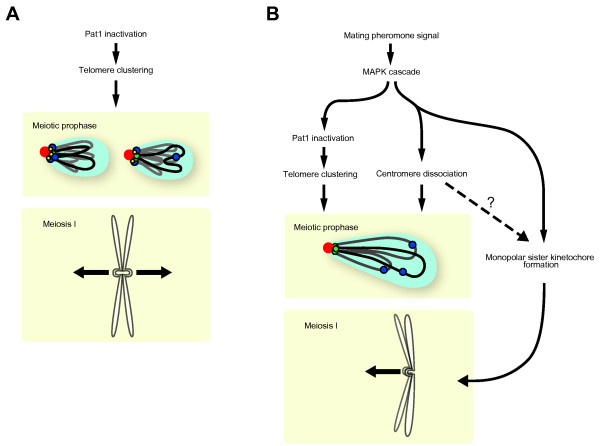
**Schematic model of nuclear organization in *S. pombe *meiosis**. (A) The nuclear organization induced by the *pat1 *mutation. When meiosis is induced by Pat1 inactivation in the absence of the mating pheromone signaling, telomeres (green) cluster at the SPB (red); however, centromeres (blue) remain associated with the SPB, and the sister kinetochores are formed in a bipolar orientation. In these cells, the NMS complex (yellow) remains at the centromere attached to the SPB. (B) Nuclear organization induced by mating pheromone signal activation. Pat1 is inactivated downstream in the mating pheromone response, leading to telomere clustering. Centromere dissociation from the SPB and formation of the monopolar sister kinetochores are regulated, probably by independent pathways, downstream of mating pheromone signaling. It remains unknown whether centromere reconstruction and/or repositioning contributes to monopolar kinetochore formation (arrow with question mark).

Regulation of meiosis is diverse among species, but the establishment of the monopolar sister kinetochore is a common and essential process in meiosis, as is the repositioning of chromosomes in meiotic prophase. Repositioning of chromosomes leading the bouquet formation promotes homologous chromosome pairing by bundling telomeres together. Although several studies indicate that repositioning of centromeres and the construction of monopolar sister kinetochores are correlated, these processes can be independently regulated in response to mating pheromone signaling (Figure 5). Although a signal pathway may be different in yeasts and higher eukaryotes, formation of the monopolar sister kinetochore is obviously crucial for meiotic chromosome segregation. How meiosis-specific proteins interact with other centromere proteins to establish monopolar sister kinetochores, and how these proteins are regulated remain to be elucidated. To address these questions, *S. pombe *provides a useful experimental system, as sister kinetochore formation can be manipulated in the presence or absence of mating pheromone signaling. Given that kinetochore components are highly conserved from yeasts to humans, we expect that analysis of the kinetochore and chromatin structures for monopolar attachment to the spindle will provide fundamental understanding of the mechanisms for ensuring the regulated segregation of homologous chromosomes and sister chromatids during meiosis.

## Competing interests

The author(s) declare that they have no competing interests.
